# The Meandering Mind: Vection and Mental Time Travel

**DOI:** 10.1371/journal.pone.0010825

**Published:** 2010-05-25

**Authors:** Lynden K. Miles, Katarzyna Karpinska, Joanne Lumsden, C. Neil Macrae

**Affiliations:** School of Psychology, University of Aberdeen, Aberdeen, United Kingdom; University College London, United Kingdom

## Abstract

**Background:**

The ability to travel mentally through time sets humans apart from many other species, yet little is known about this core cognitive capacity. In particular, what shapes the passage of the mind's journey through time? Guided by the viewpoint that higher cognitive activity can have a sensory-motor grounding, we explored the possibility that mental time travel is influenced by apparent movement through space.

**Methodology/Principal Findings:**

Participants performed a mundane vigilance task, during which they were expected to daydream, while viewing a display that elicited an illusion of self-motion (i.e., vection). Afterwards, the contents of their mind wandering experiences were probed. The results revealed that the direction of apparent motion influenced the temporal focus of mental time travel. While backward vection prompted thinking about the past, forward vection triggered a preponderance of future-oriented thoughts.

**Conclusions/Significance:**

Consistent with recent evidence that traveling mentally through time entails associated movements in space, the current results demonstrate the converse relationship—apparent movement through space influenced the temporal locus of mental activity. Together, these findings corroborate the viewpoint that mental time travel may be grounded in the embodiment of spatiotemporal information in a bidirectional manner.

## Introduction

A core facet of conscious experience is that one's mind periodically wanders from the here-and-now. From memories of lost loves to expectations about forthcoming vacations, mental time travel (MTT) makes it possible to revisit the past and pre-experience the future [1]–[5]. Present across cultures and emerging early in childhood [6]–[8], MTT is believed to serve a pivotal function in human cognition. Indeed, it has been suggested that the emergence of this ability was a critical milestone in hominid evolution [9]. When confronted with complex and challenging judgments (e.g., should I buy stocks or deposit my savings in the bank?), simulating future outcomes (i.e., *prospection*) on the basis of prior experience (i.e., *retrospection*) is a tactic that optimizes decision-making and behavioral selection [10]–[12]. That the past informs the future in this way (i.e., *recollection-guides-simulation*) is evidenced from research demonstrating that retrospection and prospection rely on largely overlapping neural structures and cognitive operations [3], [13], [14].

In addition to elucidating the neuro-anatomy of MTT [3], [14]–[17], recent work has also documented how this capacity is influenced by aging, mental illness and injury to the brain [15], [18], [19]. These important advances aside, however, remarkably little is known about the actual process of MTT and how it impacts people's behavior. In this respect, one emerging possibility is that MTT may be represented in the sensory-motor systems that regulate human movement (i.e., MTT is embodied) [20]–[22]. Characterizing the covert process of psychological time travel in this way yields some interesting behavioral predictions. Specifically, if: (i) the metaphorical arrow of time (i.e., past  =  backward, future  =  forward) [23]–[25] is grounded in a perception-action system that integrates temporal with spatial information and; (ii) embodied constructs can be revealed motorically [21], then one would expect episodes of MTT to be accompanied by distinct patterns of movement (i.e., retrospection  =  backward movement, prospection  =  forward movement). Put simply, traveling mentally in time may initiate associated bodily movements through space.

Initial evidence for such a thought-action coupling during MTT was reported in a study in which spontaneous fluctuations in the direction and magnitude of postural sway were assessed while participants engaged in either retrospective or prospective mental imagery [26]. The results revealed that the temporal locus of MTT did indeed influence the direction of people's movements — whereas retrospection was accompanied by significant backwards sway, prospection yielded postural movement in an anterior direction. Noteworthy though these findings may be, they raise important questions regarding the precise theoretical status of the sensory-motor grounding of MTT [21], [22]. In particular, it is unclear what role movement may play in effecting the higher-order cognitive activity that supports MTT. Extant accounts of embodied phenomena point to the possibility of a bi-directional relationship between mind and body [27]–[29], such that just as mental events influence bodily states, so too bodily states impact mental events. Reflecting the hypothesized grounding of MTT in sensory-motor processing [21], [22], [26], we therefore expect this latter class of effect to emerge during episodes of mind wandering. Specifically, information about one's direction of movement (i.e., backward or forward) should influence the mind's preferred temporal destination during day dreaming (i.e., past or future).

Noting that minds typically wander during tedious, easy or practiced activities [30], in the current investigation participants were required to perform a vigilance task in which to-be-detected targets appeared infrequently. Under such conditions (i.e., a cognitively undemanding task), spontaneous mental time travel was expected to occur [17]. To explore the effects of the direction of movement on the temporal locus of MTT, target items in the vigilance task were embedded in a dynamic visual display that conveyed *vection* – the illusion of self-motion [31], [32]. Vection is a common experience in daily life. Consider, for example, sitting on a stationary train and observing a carriage on an adjacent track begin to move. This situation can trigger a compelling impression that it is oneself, rather than the nearby train that is moving. Of relevance to the current inquiry, comparable sensations of self-motion can be elicited by visual displays depicting simple patterns of optical flow [33], [34]. To this end, we employed a basic star-field animation similar to that found as a screen-saver option on many personal computers. In this animation stars appeared to move either towards (i.e., centripetal inflow) or away from (i.e., centrifugal outflow) the center of the display thus inducing backward or forward linear vection, respectively [34]. Employed in this way, optical flow serves as an ideal vehicle to explore the effects of apparent self-motion in laboratory settings [35].

To summarize, participants performed a mundane vigilance task while viewing animations that specified either backward or forward vection. Afterwards, the contents of their mind wandering experiences were probed. We anticipated that the direction of optical flow would modulate the mind's preferred temporal destination during MTT. Specifically, whereas backward vection was expected to trigger participants to dwell on the past, forward vection was expected to precipitate predominantly future-oriented thoughts.

## Materials and Methods

### Ethics Statement

The study was reviewed and approved by the School of Psychology, University of Aberdeen Ethics Committee. All participants gave written informed consent prior to taking part.

### Participants and Design

Twenty-six undergraduates (aged 17–52 years; 22 females) took part in return for course credit. The experiment had a single-factor (vection: backward vs. forward) between-participants design.

### Stimuli

**Figure 1:**
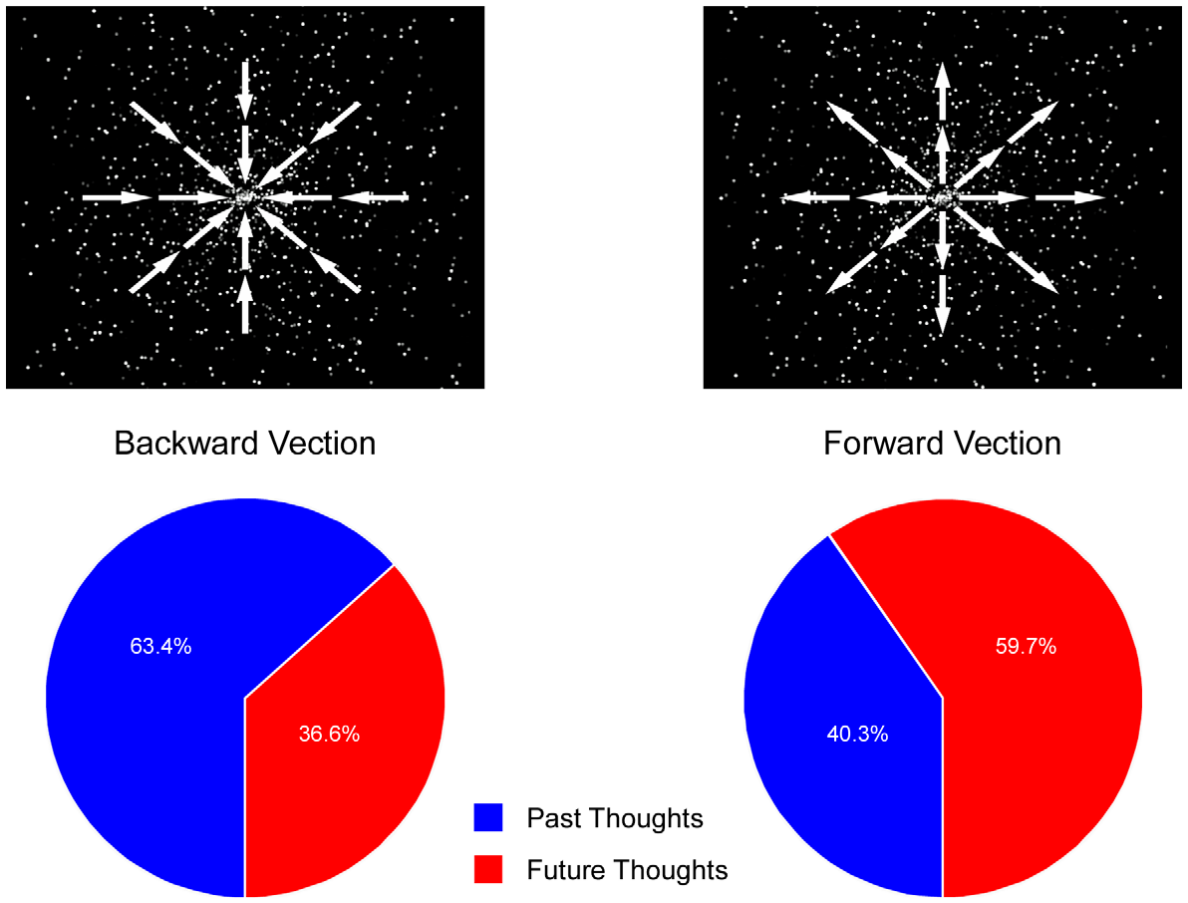
Vection and mental time travel. Illustrations of the direction of optical flow specified by the star-field displays (top panels) and the relative proportion of past and future day dreams reported by participants (bottom panels) as a function of vection condition (i.e., backward or forward).

An animated star-field display comprising approximately 1000 randomly positioned white dots on a black background was constructed (see Figure 1, top panels). The dots (i.e., stars) were animated (25 fps) so as to appear to move, on a linear trajectory, either toward (i.e., centripetally) or away from (i.e., centrifugally) the center of the display, corresponding to the experience of backward and forward vection, respectively [34]. As a manipulation check, an additional 19 participants were shown a 60 s display of either the centripetal (*n* = 10) or centrifugal (*n* = 9) star-field and asked to report the direction of any movement they experienced. As expected, participants who viewed the centripetal flow reported backward movement, while those who viewed centrifugal flow reported forward movement.

### Procedure

Participants arrived at the laboratory individually to take part in a study concerning vigilance in dynamic environments. The experimenter explained that they would be required to monitor a moving display for designated targets. Specifically, participants were instructed to click a mouse button as quickly as possible whenever they detected a target (O) but to withhold clicking when a distracter (X) appeared. Targets and distracters were super-imposed at the center of the star-field display at 3 s intervals and remained on screen for 500 ms. Importantly, targets were rare – over the course of the 6 minute vigilance task, 114 distracters but only 6 targets were presented (targets appeared at 21 s, 63 s, 162 s, 216 s, 294 s, and 333 s) thereby creating a somewhat dull task context in which day dreaming was expected to occur [30]. The display was projected onto a large screen (image size: 145 cm wide x 110 cm high) and participants were seated approximately 200 cm from the screen such that the center of the star-field was at approximately eye-level.

Immediately after completing the vigilance task, participants were asked if they had, at any stage during the procedure, experienced task-unrelated thoughts (i.e., day dreams). One participant (backwards vection condition) reported no off-task thinking and was therefore asked no further questions. The remaining participants (n = 25) were asked to estimate what proportion of their day dreams were related to past compared to future events. Specifically, they were instructed to discount any task-unrelated thoughts that were temporally located in the present and to consider only their day dreams that related to the past or the future. Participants indicated their response on an analogue scale consisting of a 150 mm horizontal line anchored by “Past Events” on the left and “Future Events” on the right. It was explained that if, for example, they had only experienced past (or future) related day dreams they should place a mark at the left (or right) extreme end of the scale, but if their off-task thoughts comprised both past and future events their mark should reflect the relative proportion of one to the other. Using the same response format, participants were also asked to rate the valence of their day dreams (anchored by “negative” and “positive”) and to report the total proportion of the time during the vigilance task they spent day dreaming (anchored by “off task” and “on task”). After completing their ratings, participants were funnel debriefed with respect to any suspicions they had regarding the purpose of the study. No participants reported any knowledge of the hypothesized link between the direction of optical flow (i.e., the direction of the star-field animation) and the temporal locus of their day dreams. Finally, participants were fully debriefed and dismissed.

## Results

The proportion of participants' day dreams relating to past compared to future events was calculated by dividing each response (measured in mm; potential range from 0 to 150) by 150 (the maximum value on the scale). The resulting scores therefore represented the proportion of task-unrelated thoughts about the future. An independent *t*-test revealed that participants in the forward vection condition reported a significantly higher proportion of future-related day dreams compared to their counterparts in the backward vection condition [*t*(23) = 2.38, *p*<.05, d = 0.94; see Figure 1, bottom panels]. No differences were observed as a function of vection condition with respect to either the valence of day dreams [*t*(23) = 0.71, *p* = .49; *M_backward_* = 64.4%, *M_forward_* = 58.4%] or the total proportion of time spent day dreaming during the vigilance task [*t*(23) = 0.68, *p* = .50; *M_backward_* = 45.4%, *M_forward_* = 39.8%].

## Discussion

The current findings reveal that the direction of vection (i.e., illusory self-motion) modulates the temporal locus of MTT. This spatial mapping of retrospection and prospection extends a rapidly emerging literature exploring both the neural correlates and representational structures involved in processing temporally-bound information [3], [13]–[17], [23]–[25], [36]. Moreover, just as it has recently been established that traveling mentally through time is associated with physical movements through space (i.e., past thoughts  =  backward movement, future thoughts  =  forward movement) [26], the current experiment demonstrated the reverse relationship – apparent movement through space influenced the temporal focus of mental activity (i.e., backward vection  =  past thoughts, forward vection  =  future thoughts). Together, these studies suggest that the core cognitive capacity of MTT may be grounded in the embodiment of spatiotemporal information in a bidirectional manner [20]–[22], [26]–[28].

While the present research adopts an embodied approach to cognition [20]–[22], [27], [28], it should be noted that more traditional information processing accounts could also be applicable. While suggestive, the current study does not provide direct evidence for the representational mechanisms that underlie the integration of time and space during episodes of MTT. That is, while theories of embodiment point to modality-specific representations of, in this case, spatiotemporal information [20]–[22], it is conceivable that such information may be stored in an amodal format (e.g., semantic networks, schemata, feature lists) [37], [38]. Similarly, it is possible that the current effects could potentially have been mediated by language to the extent that participants consciously recognized the direction of apparent motion specified by the vection display (e.g., “I feel like I'm moving backwards”), which in turn may have influenced the temporal location of their daydreams. In short, while the effects reported here are entirely consistent with a growing body of evidence demonstrating that abstract metaphorical concepts can be borne out in more concrete domains via embodied processes [20]–[22], [27]–[29], [39], [40], alternative explanations regarding the representational status of spatiotemporal information cannot, at this stage, be discounted.

The present results also draw attention to another pertinent theoretical issue – what are the foundations of the spatiotemporal coupling evident during MTT? One possibility lies in the experiential basis of people's interactions with the environment [41], [42]. By virtue of a morphology geared primarily for forward-oriented action (e.g., looking, locomoting), humans typically encounter a world that unfolds in front of them. People regulate their behavior in order that things which have yet to occur (i.e., future events) are located to the fore, whereas that which has already been (i.e., past events) is consigned to positions behind the body. This particular mapping of time and space is widely reflected in metaphoric language (e.g., looking *forward* to tomorrow, thinking *back* on yesterday), cognition [23]–[25], [36] and, as reported here, sensory-motor processing [26].

However, the conflation of abstract temporal concepts with concrete spatial information may also have more sociocultural origins. To illustrate, speakers of Aymara, an Amerindian language of the Andes, employ the reverse spatiotemporal mapping (i.e., past  =  in front, future  =  behind), during both verbal and non-verbal (i.e., gesture) communication [43]. This pattern is purportedly derived from sociolinguistic convention whereby that which is known (e.g., the past) is located to the fore, while unknown events (i.e., the future) are described as being behind the self [43]. In this way, culture (i.e., language) can play a significant role in shaping the mental integration of spatial and temporal information. Establishing which of these ostensibly contradictory hypotheses (i.e., sensory-motor vs. sociocultural experience) is most potent may be possible by investigating the developmental characteristics of space-time mappings [44], or by examining whether contrasting patterns in language (e.g., Aymara vs. English) are also revealed in strictly non-linguistic domains (e.g., movement dynamics).

One persistent challenge for researchers exploring the contents of mind wandering lies in the manner in which people's mental contents are probed [30]. Here we chose to evaluate task-unrelated thoughts retrospectively in order to avoid difficulties associated with the repeated presentation of on-line thought probes (e.g., meta-awareness of mind wandering may modulate the contents of such activity). It should be noted, however, that off-line responding can also be problematic, particularly with respect to the reconstruction of prior thoughts [45]. Reassuringly, research that has directly compared these two approaches (i.e., off-line vs. on-line experience sampling) indicates a strong consistency in the results obtained regardless of the method employed [46]–[48]. This therefore suggests that the current results accurately reflect the temporal destination of MTT. Corroboration of these findings using direct experience sampling techniques will further inform how movement impacts retrospection and prospection.

Consideration of the generalizability of the current findings raises additional points of interest. It is reasonable to question whether the effects of vection on the direction of MTT would transfer to actual physical movement. As an illusion of self-motion, vection, while compelling, lacks the richness of information (e.g., proprioceptive, haptic or auditory information) that regularly accompanies movement. Thus, as a relatively impoverished sense of motion, vection may be considered a somewhat conservative test of the hypothesized relationship between the direction of movement and the temporal locus of MTT. In this sense, it is possible that the current effects would be amplified if participants were required to physically move. Indeed, as noted previously, in naturalistic settings people tend to move forward through their environment. What this therefore suggests is that spontaneous MTT may be focused primarily on future events [9], [11], a prediction that finds support in the existing literature on daydreaming [49], [50]. Establishing exactly how and why space-time mapping impacts social-cognitive functioning remains an important task for future research.
